# Equine Stomach Development in the Fetal Period: An Anatomical, Topographical, and Morphometric Study

**DOI:** 10.3390/ani12212966

**Published:** 2022-10-28

**Authors:** Dominik Poradowski, Aleksander Chrószcz

**Affiliations:** Department of Biostructure and Animal Physiology, Division of Animal Anatomy, Faculty of Veterinary Medicine, Wroclaw University of Environmental and Life Sciences, Kożuchowska 1, 51-631 Wrocław, Poland

**Keywords:** equine, stomach, development, fetal period, anatomy, topography, morphometry

## Abstract

**Simple Summary:**

The prenatal stage of life can be divided into embryonic and fetal periods. While the course of the embryonic period has been investigated to some degree, the fetal period has been described only in general. The details of stomach development in the fetal period are unknown in any domesticated mammals excluding swine. This study describes changes in equine stomach morphology, shape, and location between the 4th and 11th months of pregnancy. The equine stomach with all its anatomical parts is already developed at four months of gestation. During further development, the stomach dimensions change and the shape of the stomach evolves from medium-wide to wide and from slightly bent to strongly bent. The gastric mucosa become differentiated as the fetal period progresses, forming typical anatomical features. Our statistical analysis of the majority of the metric parameters revealed that their growth rate was faster than the increase in crown–rump length. This study proved that dynamic development of the equine stomach can be traced not only in the embryo but also in the fetus. In this way, the study fills the knowledge gap concerning this part of prenatal life in horses.

**Abstract:**

Studies of equine stomach prenatal development are very rare, and descriptions usually focus on the processes taking place in the embryonic period. Only general information about gastric organogenesis in the fetal period is available in embryology textbooks on domestic mammals. The material for our study included twenty half-breed horse fetuses divided into three age groups on the basis of known fetal age (verified using the CRL method). Our study consists of the topographical, morphological, and morphometrical description of stomach development between the 4th and 11th months of gestation. Even though the skeletotopy, syntopy, and holotopy of the stomach in the fetal period seems to be relatively unchanged, the organ shape and the proportions between its anatomical parts differed in fetuses from the three age groups. The achieved results were statistically elaborated to estimate the dynamics of the stomach shape. This can be described as changing from medium-wide to wide and from slightly bent to sharply bent. A nonlinear correlation of all metric values with CRL in all age groups was observed. A positive allometric growth rate of different intensity was seen in all metric parameters. All the values increased as the fetal period progressed. Only the parietal surface growth rate gradually changed from strongly positive allometric in the first age group to strongly negative allometric in the third age group. A difference between the non-glandular and glandular mucosa of the stomach was visible in the first group. Development of a well-distinguishable plicated edge margin began in the second age group together with gastric pits and gastric areas. The third age group showed a well-developed gastric groove and angular incisura.

## 1. Introduction

Intravital and noninvasive imaging methods, routinely used in clinical practice, can be useful in studies of prenatal life, especially of the fetal period [1,2]. Good examples of sonography incorporation into prenatal investigations include morphological analyses of the orbit, carotid artery, and intestine in equine fetuses [3,4,5]. A detailed embryological study of equine stomach development in the fetal period seems a valuable contribution to interdisciplinary studies integrating both basic and clinical veterinary sciences.

Even though the prenatal development of mammals has been frequently described in the research literature, the studies usually target embryogenesis and the embryonic period [6,7,8]. Equine prenatal development between the 15th and 107th day of gestation was reported in detail by Franciolli et al. [9], but studies of the fetal period and of the development of the foregut caudal part (forming the stomach and gastric wall structures) are rare in the literature. The majority of researchers have focused on laboratory animals, as they are more accessible for such investigations [10,11,12,13,14,15,16,17,18,19,20]. Only a few studies have been aimed at the prenatal development of the stomach in domestic mammals [11,12,13,14,15,16,17,18,19,20,21,22,23,24,25,26,27,28]. The lack of a detailed description of horse stomach prenatal development in the fetal period inspired us to undertake an anatomical and histological analysis of this organ. 

Chrószcz studied the morphometry and topography of a swine stomach in the fetal period between the 35th and 114th day of gestation [25] and described in detail the development of the stomach wall and its structures [26]. Both papers broadened our knowledge of swine stomach development in the fetal period and are good examples of investigations that have informed further studies of horse fetuses.

According to Marrable [29], the prenatal stage of life can be divided into embryonic and fetal periods, with the turning point being sexual differentiation and gonad development. The exact boundary between these stages of prenatal development is not sharp, but at the beginning of the second stage organogenesis is finished and all the parts of the skeleton are distinguishable. The fetal period in horses starts on the 40th day of gestation, even though the sex of the fetus is not distinguishable until the 47th day of gestation [9]. The terminal part of the foregut is initially a simple tube, and fusiform enlargement occurs on the 25th day of gestation, leading to the formation of a primordial stomach [9,28]. During the stomach rotation, the organ is placed in its final position transverse to the long axis of the body. On the 30th day of gestation, the stomach wall is differentiated into the mucous membrane, submucosa, muscular layer, and serosa [28].

The stomach of an adult horse is morphologically divided into a non-glandular part (*saccus caecus*) covered with stratified squamous epithelium (epithelium multistratificatum planum) and a glandular part, which are separated by plicated edge margin (*margo plicatus*). In general, the equine stomach can be classified as a complex stomach, intermediate between a monogastric and a polygastric stomach [30]. The fetal stomach without gastric glands can be observed on the 35th day of gestation, and both dorsal and ventral gastric mesenteries forming the greater and the lesser omentum are visible on the 50th day of gestation [28]. The development of adjacent abdominal organs, such as the liver, intestines, and spleen, must correlate with the development of the gastric primordium [6,7,8,29]. Rapid development of the liver in the early fetal period gives way to stomach and intestine development in the middle fetal period. These mechanisms of mutual interaction have been described for the prenatal life of a swine fetus [25] but remain unknown in other domestic mammals, including horses. 

A comparison of the allometric and isometric phases of abdominal organ growth in the fetal period would be especially interesting. The aim of this study was the morphological and morphometric analysis of stomach development in the fetal period, which allowed for recognizing changes in the organ proportions and shape as well as for assessing the stomach wall growth dynamics in the allometric and isometric phases of the fetal period.

## 2. Materials and Methods

### 2.1. Material Preservation and Age Estimation

The material used in this study consisted of 20 half-breed horse fetuses at the 4th to the 11th month of gestation preserved in the morphological collection at the Division of Animal Anatomy at the Wroclaw University of Environmental and Life Sciences. All the fetuses were preserved with continuous intraarterial injections (umbilical vein) of 4% paraformaldehyde. Next, the material was stored in 4% paraformaldehyde and rinsed with running water before the morphological analysis. The adult equine stomachs were collected in a slaughterhouse. As the tissue samples were not taken from alive animals, approval of an ethics committee was not necessary.

The age of the fetuses was known due to the mare’s history of pregnancy and the day of miscarriage. The age of the fetuses was verified using the crown–rump length (CRL) method [28,29,31,32]. The material was divided into three age groups according to the population cross-section method [25,26,27,33]. The first age group consisted of fetuses at 4–5 months of gestation (*n* = 5), the second of fetuses at 7–8 months (*n* = 5), and the third of fetuses at 10–11 months (*n* = 5). Each age group consisted of four fetuses.

The fourth group of adult animals (5–8 years old) was added as a reference to compare the features of a fully developed stomach wall with developmental stages of the morphological structures in the fetal period.

The accessible material was preserved in buffered 4% paraformaldehyde for two to three months. Before dissection, each fetus was rinsed in running water and the CRL was measured and compared with referential values.

### 2.2. Morphological Analysis

The anatomical analysis of all the equine fetuses investigated in this study followed the protocol below:

- fetus dissection and anatomical preparation of the stomach (laparotomy in the median and transverse line),
- fetus gender identification based on external and internal genital organs,
- topographic analysis of the cranial abdominal region and the stomach location (holotopy, skeletotopy, and syntopy) according to the methods introduced by Chrószcz [25].

### 2.3. Morphometric Analysis

**Figure 1:**
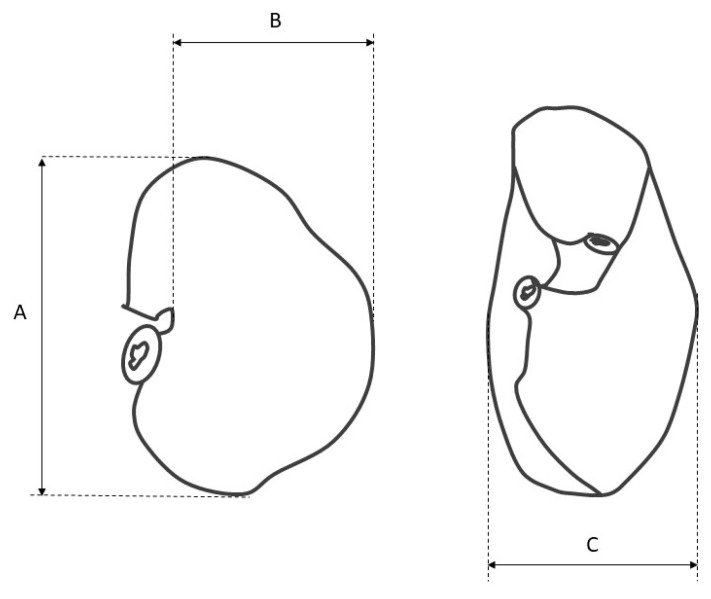
The scheme of the stomach morphometry. A—the length of the stomach; B—the width of the stomach; C—the thickness of the stomach.

The morphometry of the stomach was carried out according to Stelmasiak et al. [34] and Biedermann [35]. Each measurement was performed with an electronic slide caliper and repeated three times; the mean value was computed. The morphometric analysis included the following steps:

- measurement of the stomach filled with gelatin to estimate its length, width, thickness, and volume (Figure 1),
- measurement of the length of the greater and lesser curvature,
- measurement of the diameter of the cardiac and pyloric orifice,
- gastrotomy along the greater curvature, and measurement of the distance from the cardiac orifice to the fundus and from the cardiac orifice to the greater curvature (along the parietal and visceral surface),
- calculation of the width/length index and the curvature index to recognize the shape of the stomach changes during subsequent phases of the fetal period [20,29].

The description of macro- and microanatomical findings was carried out with Nomina Histologica Veterinaria [36], Nomina Anatomica Veterinaria [37], and Nomina Embryologica Veterinaria [38].

### 2.4. Statistical Analysis

OriginPro package, version 2021 (OriginLab Corporation, Northampton, MA, USA) was used for the statistical analysis. The data obtained for individual parameters were averaged, and the standard deviation (SD) was calculated. The regression curves were made using the Simple Fit application in OriginPro. On the basis of the results plotted on the diagrams, linear and nonlinear regression curves (allometric and polynomial approximation) were obtained. The Pearson’s correlation coefficient (r) was used as a measure of linear fit, where |r| = 1 means the strongest association of two variables. The correlation coefficient (R^2^ COD) was used as a measure of nonlinear curve fit, where R^2^ = 1 means that the fitted line explains all variability in the response data around its mean. All the results are presented in figures, tables, and diagrams.

## 3. Results

### 3.1. CRL Analysis

**Figure 2:**
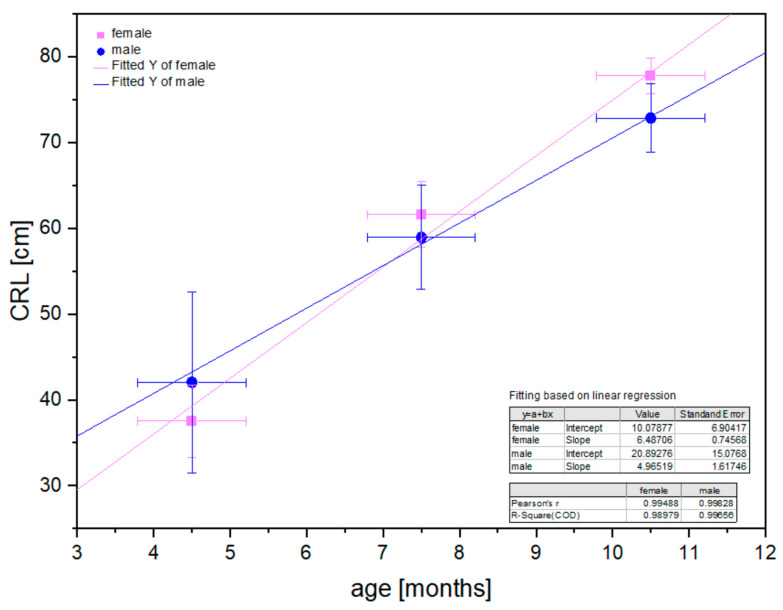
Linear regression in CRL values in the fetal period for male and female horse fetuses.

The statistical analysis of the CRL estimated in all age groups indicated a linear correlation with the fetus age both for male and female fetuses (Figure 2).

### 3.2. Anatomy and Topography of the Stomach (Holotopy, Skeletotopy, Syntopy)

In all the analyzed fetuses, the stomach was located in the cranial abdominal region. Table 1 shows a skeletotopy of the organ.

The values ranged from 11 to 17 intercostal space. The cardiac orifice was located at the transverse body cross-section, specifically at the 12th intercostal space (on the border between the dorsal and medial, one-third of the median plane of the animal body), and the pyloric orifice was located in the right hypochondriac region at the 13th to 14th intercostal space (on the border between the dorsal and ventral half of the cranial abdominal region). 

The cardiac part of the stomach surrounded the cardiac orifice within the cranial wall of the developing blind ventricular sac (the second and third age groups). The body of the stomach was located in the left hypochondriac region, and the blind ventricular sac was clearly distinguishable in the second age group. The pyloric part of the stomach was located in the right body side, near the portal of the liver, joining the cranial part of the duodenum. The caudal border of the stomach did not extend to the border indicated by the course of the costal arch. 

The liver with five distinguished lobes surrounded the stomach cranially, dorsally, laterally, and ventrally. The parietal surface of the stomach (blind ventricular sac and body of the stomach) adhered directly to the left lobes and quadrate lobe of the liver (the gastric impression). The pyloric part of the stomach was located caudally from the border between the quadrate and right lobe of the liver. The fundus of the stomach was located ventrally and cranially to the left kidney. Near the lesser curvature, the visceral surface of the stomach adhered to the pancreas. 

The gastric ventral mesentery, attached to the lesser curvature of the stomach, was divided into two parts: the hepatogastric ligament and the falciform, coronary, and triangular ligaments of the liver. The greater curvature of the stomach was the attachment point of the gastrolienal ligament (part of the greater omentum). The spleen was triangular and located longitudinally to the greater curvature of the stomach, to the left, laterally and caudally from the stomach. The right kidney was located dorsally and caudally from the stomach and pancreas, in direct contact with the liver caudate lobe (the renal impression). The visceral surface of the stomach adhered to the diaphragmatic flexure of the right and left dorsal colon (the large colon), to the loops of the small intestine dorsally (the median plane), and to the transverse colon. The sternal flexure of the left and right ventral colon and the apex of the caecum were located ventrally from the stomach on the abdominal floor.

**Figure 3:**
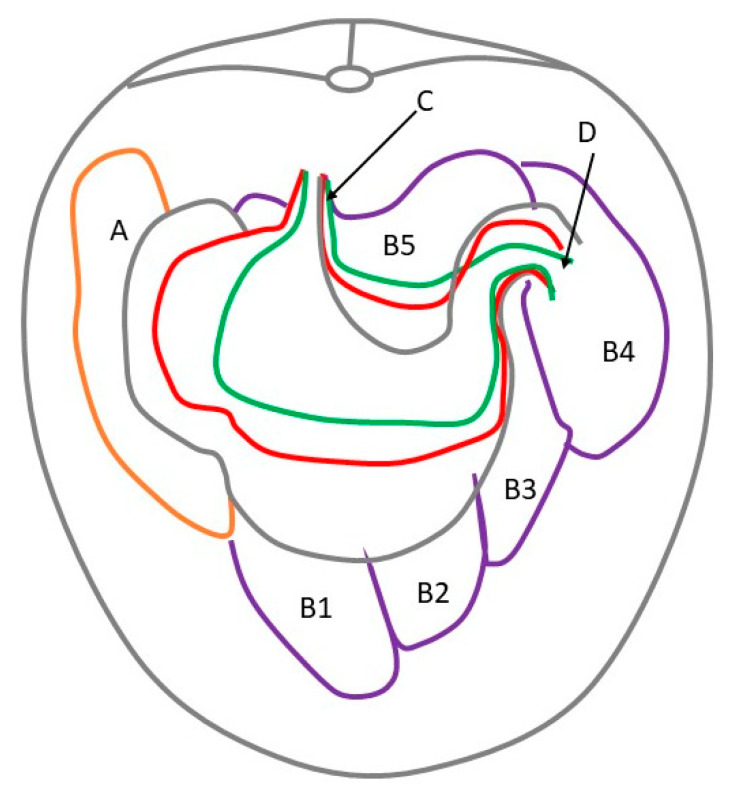
Topography of the stomach in the fetal period (caudal view). Green—first age group; red—second age group; black—third age group; A—spleen; B1—liver, left lateral lobe; B2—liver, left medial lobe; B3—liver, quadrate lobe; B4—liver, right lobe; B5—liver, caudate lobe; C—cardiac orifice; D—pyloric orifice.

**Figure 4:**
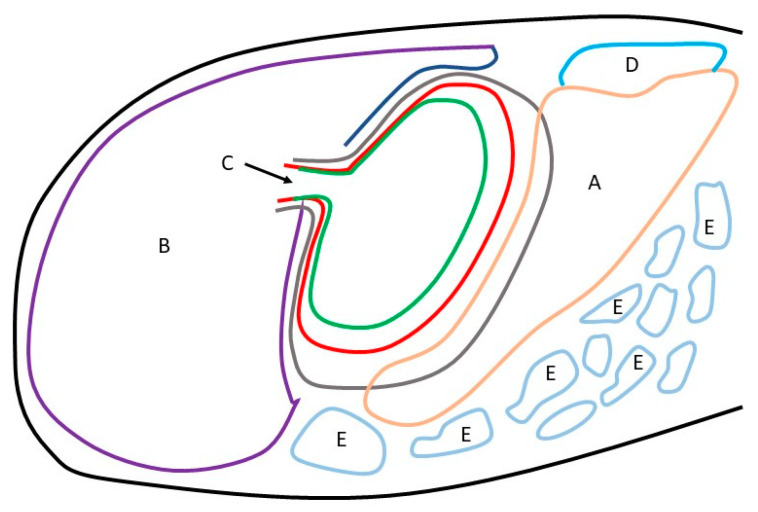
Topography of the stomach in the fetal period (lateral view). Green—first age group; red—second age group; black—third age group; A—spleen; B—liver; C—cardiac orifice; D—kidney; E—small and large intestine.

During the prenatal development between the 4th and 11th months of gestation, the most visible changes included the position of the greater curvature of the stomach and the location of the liver ventral border. In the first age group, a well-developed liver was the most prominent organ of the cranial abdominal region. A small, bent gastric sac was inserted in the left hypochondriac region between the left lobe of the liver and the developing small and large intestines. The most ventral point of the greater curvature reached to the half of the abdominal cavity height. In the second age group, the developing stomach changed the greater curvature’s position and reached the border between the ventral and middle one-third of the abdominal cavity height, which remained constant in the third age group. The developing blind ventricular sac reached the 14th to the 15th intercostal space (Figure 3 and Figure 4).

### 3.3. Morphometry of the Stomach

The metric analysis of the stomach assessed its length, width, thickness, cardiac and pyloric orifice diameter, depth of the blind ventricular sac, distance between the cardiac orifice and the right and left margin of the greater curvature, and length of the greater and lesser curvature (Table 2).

Based on the measurements, the shape of the stomach in all investigated age groups was defined using gastric indexes (Table 3).

**Figure 5:**
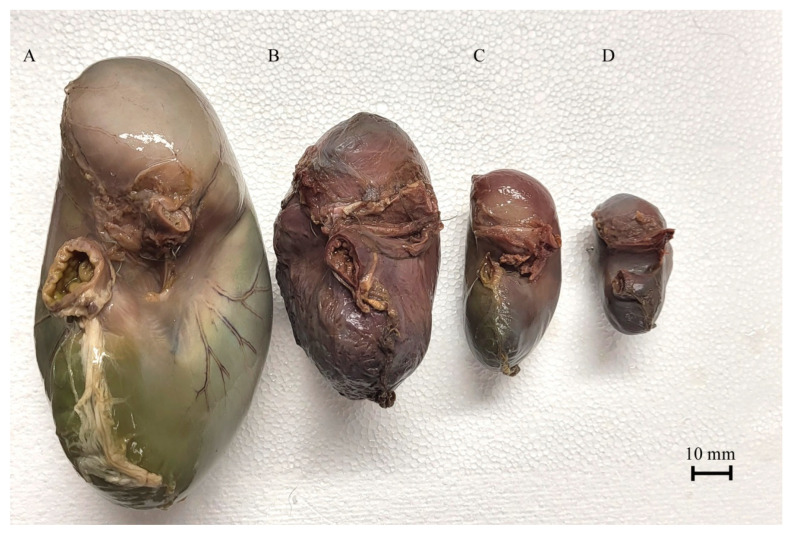
Equine fetal stomach (the lesser curvature view). A—the third age group, B—the second age group, C and D—the first age group.

**Figure 6:**
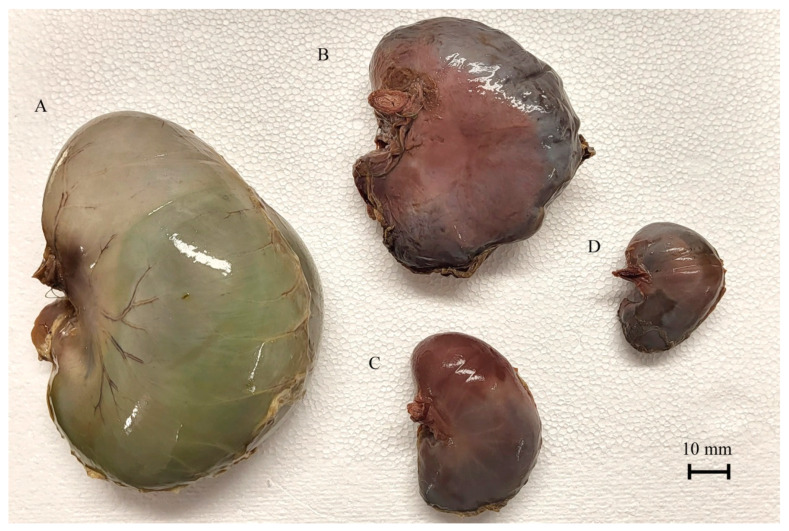
Equine fetal stomach (the visceral surface view). A—the third age group, B—the second age group, C and D—the first age group.

The exact shape of the filled stomach was visualized using a gelatin injection into the stomach lumen (Figure 5 and Figure 6).

The blind ventricular sac of the stomach was weakly distinguishable even in the first age group. In the second age group, the cardiac orifice was located in the ventral part of the developing blind ventricular sac, which was clearly separated from the glandular stomach in the third age group. The parietal surface of the stomach was slightly convex (most prominently in the second age group) as compared with the visceral surface, which protruded strongly caudally especially in the first and third age groups. The distance between the cardiac and pyloric orifice was small, and the factor most influencing the shape of the stomach was the length of the greater curvature.

**Figure 7:**
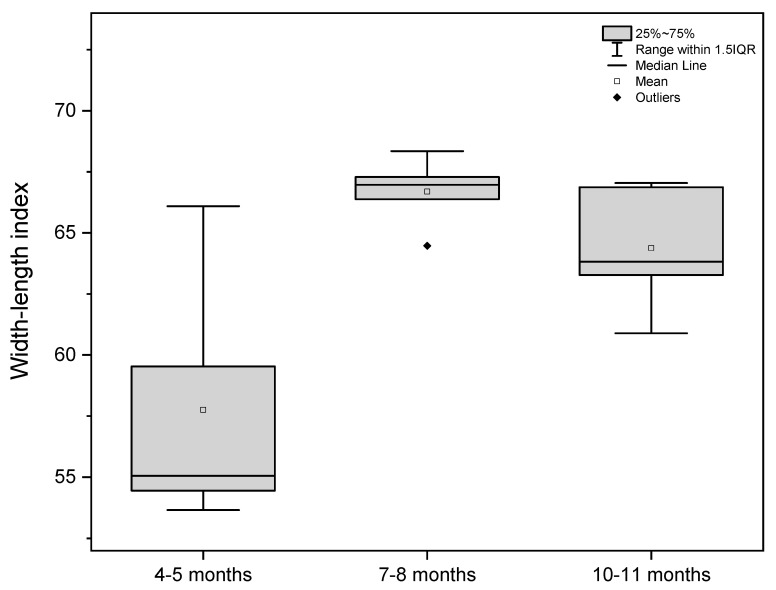
Width-length index values in the fetal period (4th to 11th month of gestation).

**Figure 8:**
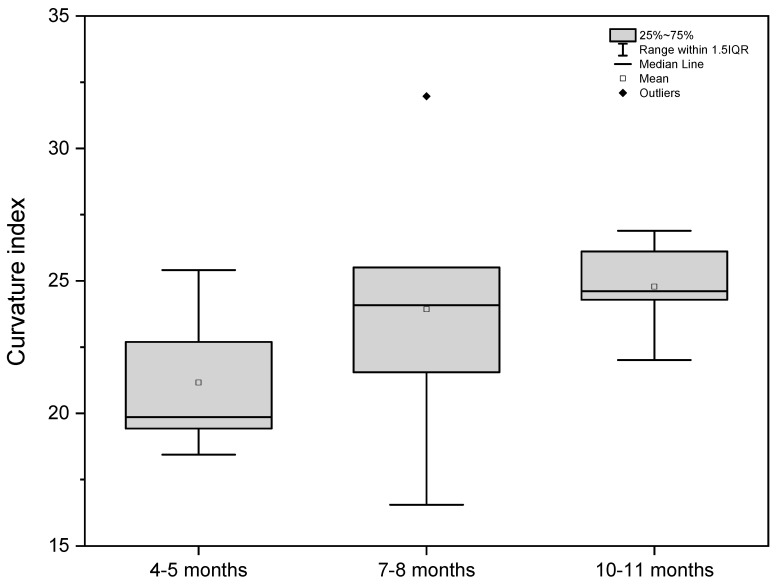
Curvature index values in the fetal period (4th to 11th month of gestation).

The stomach indexes were computed on the basis of organ length, width, and thickness and the length of the greater and lesser curvature. The width–length index and the curvature index were chosen as the most important indicators for stomach shape estimation. The statistical analysis of the above-mentioned indexes is presented as box-plots (Figure 7 and Figure 8).

The width–length index in the first age group classified the stomach shape as medium-wide. The same index in the next two age groups described the organ shape as wide. Simultaneously, the curvature index analysis indicated that the stomach shape changed from slightly bent (first age group) to sharply bent (second and third age groups). 

**Figure 9:**
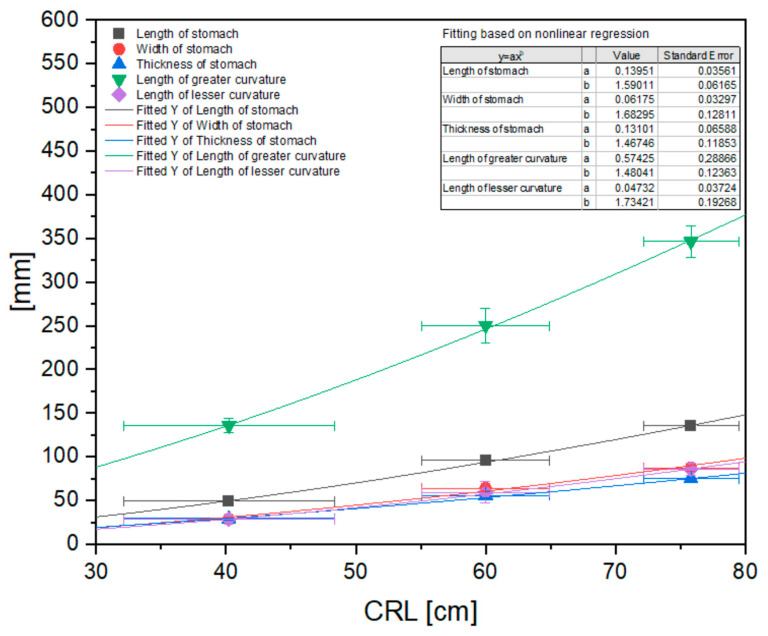
Nonlinear regression of basic stomach morphometric values (length, width, and thickness, and length of the greater and lesser curvature). CRL—crown–rump length.

**Figure 10:**
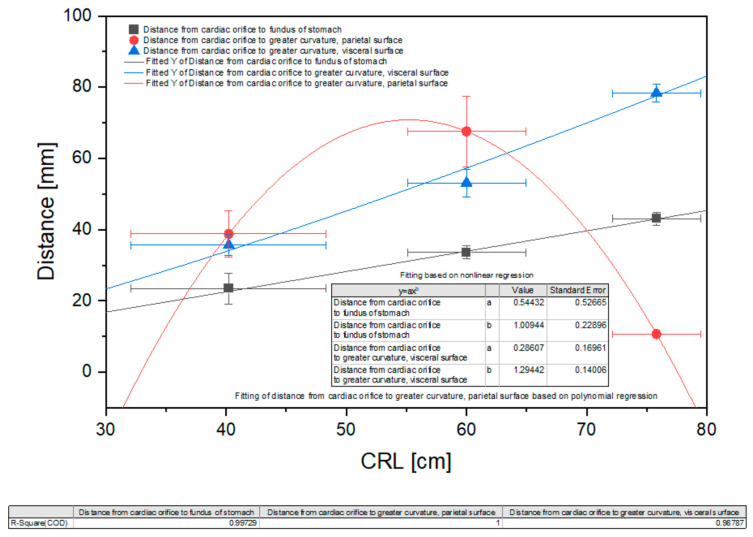
Nonlinear regression of the stomach morphometric values (distance from the cardiac orifice to the stomach fundus, from the cardiac orifice to the greater curvature and the parietal surface, and distance from the cardiac orifice to the greater curvature and the visceral surface). CRL—crown-rump length.

**Figure 11:**
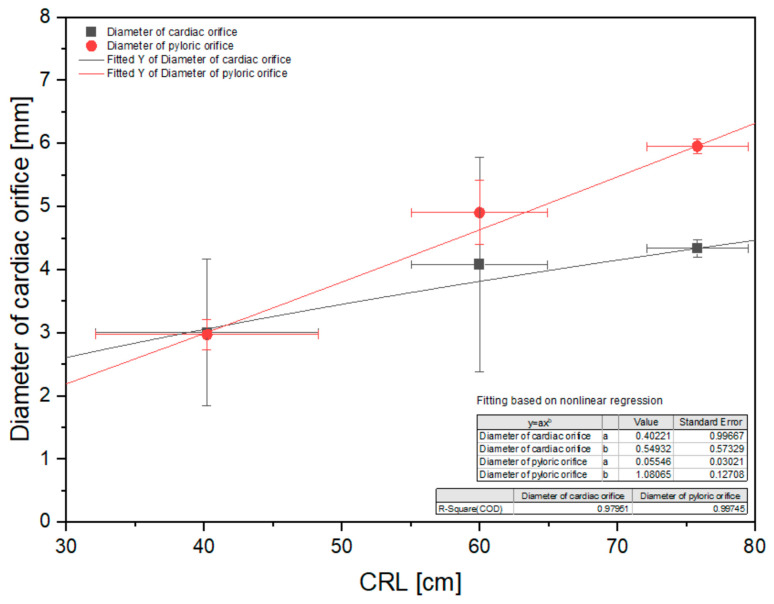
Nonlinear regression of the stomach morphometric values (diameter of the cardiac orifice and pyloric orifice). CRL—crown–rump length.

The morphometric measurements of the stomach were statistically analyzed and are presented in Figure 9, Figure 10 and Figure 11.

The statistical analysis of the measurements revealed a nonlinear correlation of all values with CRL in all age groups. This allowed us to compare the allometric growth of the mentioned parameters with the isometric character of the CRL growth in the fetal period. The most intense positive allometric growth was observed for the length of the greater curvature, and for the other parameters (stomach length, width, thickness, and length of the lesser curvature) the positive allometric growth was weaker. In all cases, the values of the measured parameters increased as the pregnancy (the fetal period) progressed (Figure 9).

The growth rate of the distance from the cardiac orifice to the stomach fundus (depth of the blind ventricular sac) showed a slightly positive allometric character. More evident positive allometric growth was visible for the distance from the cardiac orifice to the greater curvature (width of the gastric wall along the visceral surface). Finally, the growth rate of opposite gastric wall width (distance from the cardiac orifice to the greater curvature and the parietal surface) can be described as strongly positive allometric in the first age group, gradually decreasing in the second age group, and strongly negative allometric in the third age group, as defined by the polynomial curve visible in Figure 10.

The diameter of the cardiac orifice showed a slightly negative allometric growth, and the diameter of the pyloric orifice can be characterized as growing in a slightly negative allometric way (Figure 11).

### 3.4. Stomach Mucosa Macroanatomy

**Figure 12:**
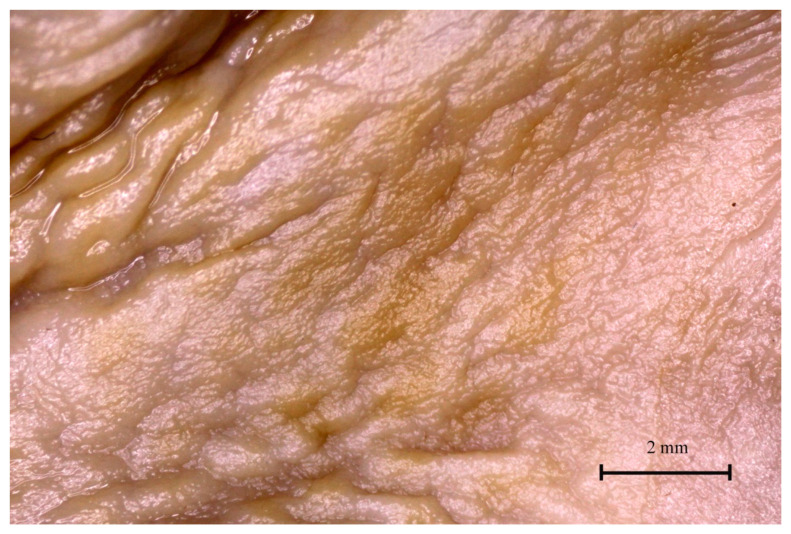
Gastric mucosa in the plicated edge margin region (the first age group).

The mucosa of the stomach observed under a stereoscopic microscope (Zeiss Stemi 2000-C microscope, 1.6×) differed significantly in all three age groups. The border between the non-glandular and glandular part of the stomach (plicated edge margin) was not visible in the first age group (Figure 12).

**Figure 13:**
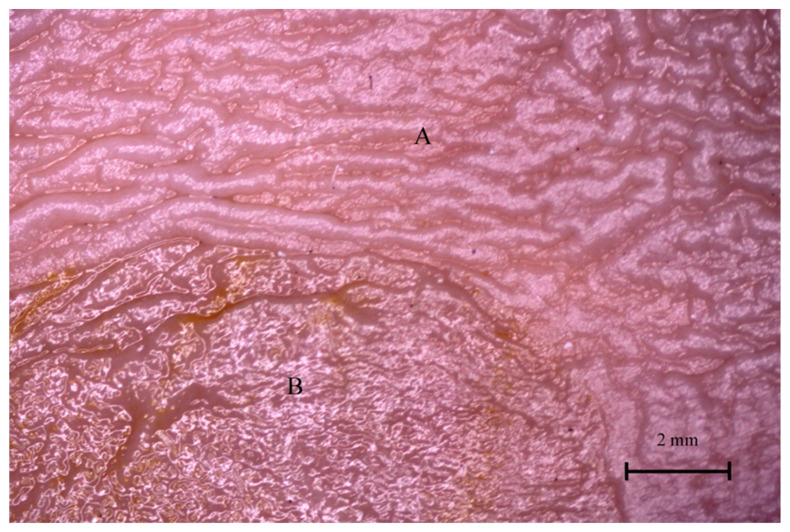
Gastric mucosa in the plicated edge margin region (the second age group). A—non-glandular part, B—glandular part.

**Figure 14:**
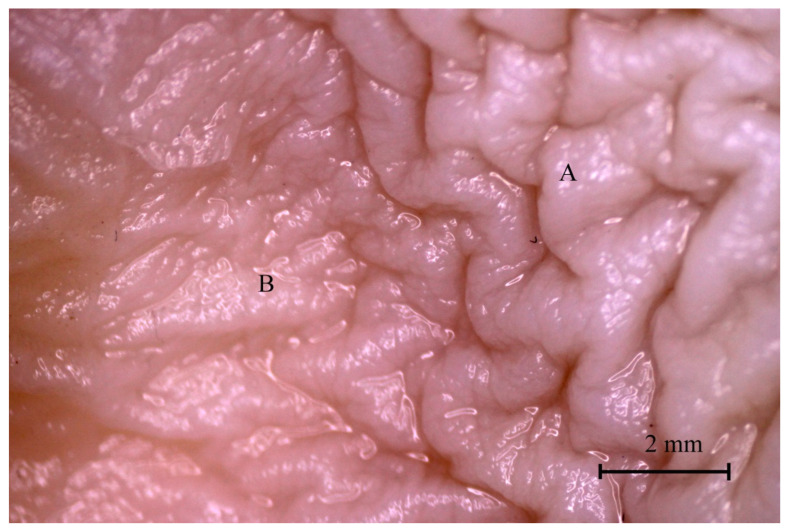
Gastric mucosa in the plicated edge margin region (the third age group). A—non-glandular part, B—glandular part.

The difference between both parts of the stomach was distinguishable in the second age group and well visible in the third age group (Figure 13 and Figure 14).

**Figure 15:**
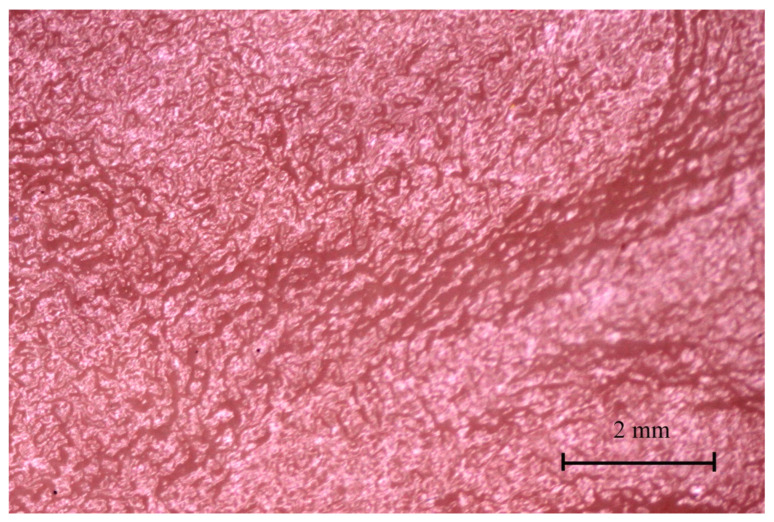
Gastric mucosa of the stomach body (third age group).

The gastric areas and gastric pits of the glandular part were observed in the second and third age groups (Figure 15). The third age group also showed a well-developed gastric groove and an angular incisura.

## 4. Discussion

The prenatal stage of life can be divided into the first phase of ontogeny (the embryonic period), and the second phase, when the primordia of the internal organs have advanced in their development and the majority of the morphological changes consist in the process of final organ positioning and growth allowing for sufficient resumption of physiological function after birth [6,7,8,29]. Anatomical and embryological studies of the fetal period are rare. Excluding some general information on domestic mammals in embryology textbooks, a full description of stomach development in the fetal period of the pig was first introduced by Chrószcz [25]. Even though the crucial work devoted to swine embryology [29] described the prenatal development of the pig stomach, it focused mainly on the embryonic period. 

The morphometric studies of the stomach in adult horses carried out by Biedermann [35] gave an important basis for embryological studies of organ development in swine [25] and in the fetal period of horses. Equine stomach development was described only between the 15th and 107th day of gestation [9]; therefore, studies investigating the fetal period have been urgently needed.

The sex of the fetus was analyzed as the factor influencing the prenatal development of selected structures [25,26,27,39,40]. All these publications proved that morphological differences were not associated with the sex of the fetus. This study analyzed the basic value (CRL) describing the prenatal development of the fetus and demonstrated a linear correlation of CRL with the fetus age group and nonstatistical differences between male and female fetuses (Figure 2). Similar results were obtained for CRL analysis carried out in pig fetuses [30,31]. In summary, this assumption can be extrapolated to the other metric parameters considered in this study.

The 40th day of gestation marks the beginning of the fetal period. The position of the stomach is similar to that observed in postnatal life after the 30th day of pregnancy [28]. The position and topography of the stomach in the first age group (4th to 5th month of gestation) proved that the most important changes in stomach morphology involved differences in the organ proportions in subsequent phases of the fetal period (second and third age group) (Figure 3 and Figure 4). The morphometry of the equine fetal stomach (length, width, and greater and lesser curvature length) characterized the organ as slightly bent and medium-wide in the first age group, and sharply bent and wide with significant blind ventricular sac (left) and strong pyloric part (right) in the second and third age groups (Figure 5 and Figure 6). The same parameters when used to describe a pig fetal stomach (Figure 7, Figure 8 and Figure 9) characterized it as medium-wide and slightly bent [25]. This can be explained by the fact that fewer significant morphological changes occurred during the ventricular diverticulum development in pigs than during the blind ventricular sac formation as the stomach semi-compartment in horses. The fetal liver was positioned constantly in the cranial abdominal region in both pigs and horses (Figure 3 and Figure 4). This is the most cranially located organ of the abdominal cavity and separates the stomach from the diaphragm. The concave visceral surface of the liver in the prenatal life of a horse influences the shape of the stomach. This study proved that the most convex shape of the parietal surface of the stomach was observed in the second age group (Figure 10). This phenomenon was probably caused by a slowing down of the positive allometric growth of the liver and its transition into isometric and negative allometric growth in the second stage of pregnancy. The rate of stomach visceral surface growth is positive allometric and influenced by the development of the small and large intestine (Figure 10). The decrease in growth rate of the stomach parietal surface to the negative allometric growth in the third age group seemed to be caused not by intensified positive allometric growth of the liver (it remained constantly isometric until birth) but by intense colon pressure resulting in the flattening of the stomach wall adjacent to the solid organs such as the liver. Moreover, the constant positive allometric growth of the blind ventricular sac corresponded with a decrease in the liver growth rate. Similar changes were much less significant in swine fetal stomach development (Chrószcz 2008, [25]).

Even though the skeletotopy and syntopy of the stomach were established, the shape of the organ showed significant changes in the length of the greater curvature. Its growth was described as significantly more positively allometric than in the other basic morphometric parameters of the stomach (length, width and thickness, and length of the lesser curvature) (Figure 9). These parameters also showed positive allometric growth, but less significant than that of the length of the greater curvature. This can be explained by the stomach morphology typical in horses in postnatal life, i.e., a sharply bent stomach with an angular incisure. The stable location of the cardiac and pyloric orifices, together with slow growth (positive allomeric) of the lesser curvature allows for the stomach transition from slightly bent to sharply bent shape with an increase in the length of the greater curvature that achieves its final, most ventral position in the third age group.

Finally, the stomach mucosa surface showed significant differences in the first and third age groups. The plicated edge margin, forming the border between the non-glandular and glandular parts of the stomach, was visible in the second age group (the most convex parietal surface of the stomach) (Figure 12, Figure 13 and Figure 14). In the same age group, the positive allometric growth of the parietal surface slowed down and switched to negative allometric growth in the third age group (Figure 10). The gastric areas and the gastric pits typical for stomach glandular mucosa were visible in the second age group (Figure 15). This corresponded with the development of the gastric glands in the second half of gestation in pigs [26]. The detailed description of gastric mucosa development will be the aim of a separate paper.

Changes in the diameter of the cardiac and pyloric orifices can be influenced by the development of the strongest muscular layer of the stomach wall around its pyloric orifice [6,7]. This resulted in a stronger growth rate (positive allometric in both cases) manifested in the pyloric orifice diameter (Figure 11). The cardiac orifice diameter can be influenced by the physiological sphincter of cardia development from the stomach muscular layer, which is typical for horses.

**Figure 16:**
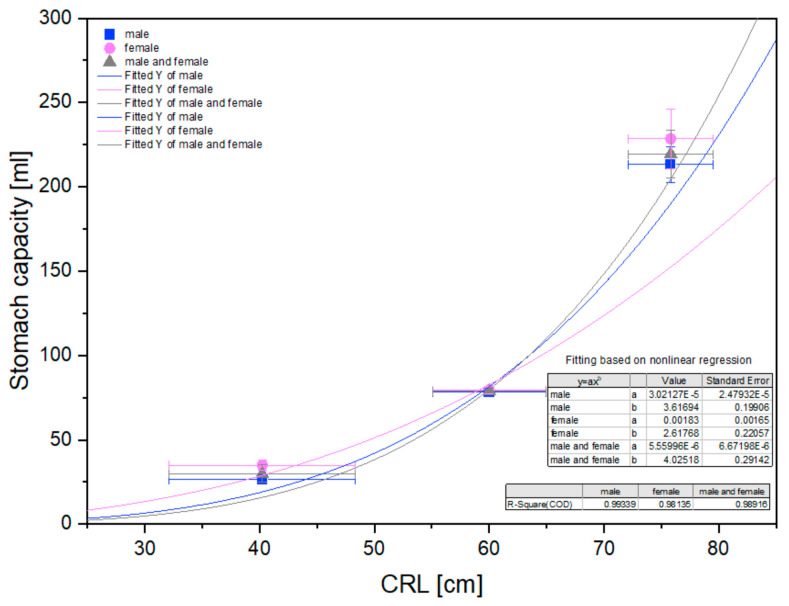
Stomach volume in subsequent age groups in relation to CRL.

The stomach capacity showed a positive allometric growth rate, which correlated with an increase in the organ morphometric parameters in males, females, and both sexes analyzed together (Figure 16).

## 5. Conclusions

In conclusion, the stomach is the digestive tract compartment responsible for ingested food storage and initiation of digestion and which must be able to fill its physiological function directly after birth. All the analyzed parameters proved that during the fetal period stomach morphology undergoes significant changes, presented here as statistical elaborations of the results. The fetal period is characterized by a positive allometric growth rate of the stomach morphometric parameters that can differ in intensity. The clear differentiation into non-glandular and glandular parts with developed plicated edge margin, gastric pits, and gastric areas was visible in the second age group. The stomach shape was classified as medium-wide to wide and as slightly bent to sharply bent with the angular incisura and the gastric groove clearly distinguishable in the third age group. Further studies of the gastric embryology using intravital sonography would be useful to compare the presented results with those in live fetuses. Such a comparison would be important not only for developmental anatomy but also for equine obstetricians and perinatologists.

## Figures and Tables

**Table 1:** Skeletotopy of the horse stomach.

Sex	M	M	M	F	F	M	M	M	F	F	M	M	F	F
Age [months]	4–5	4–5	4–5	4–5	4–5	7–8	7–8	7–8	7–8	10–11	10–11	10–11	10–11	10–11
Intercostal space	14–17	13–16	14–16	14–17	14–16	13–16	14–16	12–16	13–17	12–16	13–16	12–16	11–16	12–16

**Table 2:** Morphometry of the horse stomach.

Sex	M	M	M	F	F	M	M	M	F	F	M	M	F	F	F
Age [months]	4–5	4–5	4–5	4–5	4–5	7–8	7–8	7–8	7–8	7–8	10–11	10–11	10–11	10–11	10–11
Stomach length [mm]	50.46	51.65	49.11	48.25	47.68	95.65	92.41	98.42	91.49	102.3	136.11	133.34	137.11	139.25	131.98
Stomach width [mm]	30.04	27.71	26.74	31.89	26.25	64.36	61.89	63.45	62.53	67.91	91.02	89.39	83.49	88.11	84.23
Stomach thickness [mm]	29.51	26.82	30.84	31.64	27.23	54.14	53.16	58.56	51.56	62.11	72.14	74.54	77.72	74.39	75.43
Length of greater curvature [mm]	141	141	139	136	122	284	245	244	232	247	357	337	368	350	321
Length of lesser curvature [mm]	26	32	27	27	31	47	59	78	50	63	96	88	81	85	79
Diameter of cardiac orifice [mm]	2.15	4.83	2.23	2.32	3.49	2.62	6.15	5.32	2.16	4.15	4.32	4.11	4.36	4.51	4.37
Diameter of pyloric orifice [mm]	3.38	2.75	2.88	2.95	2.89	5.53	4.17	5.01	5.13	4.68	6.13	5.82	5.92	6.01	5.88
Distance from cardiac orifice to stomach fundus [mm]	17.28	26.17	21.32	28.39	24.34	33.69	36.35	32.59	31.38	34.32	40.38	42.39	44.13	45.23	43.45
Distance from cardiac orifice to greater curvature parietal surface [mm]	29.61	36.17	43.11	46.39	38.72	78.57	77.49	61.21	56.46	64.32	9.84	11.39	10.01	11.93	10.32
Distance from cardiac orifice to greater curvature visceral surface [mm]	34.17	31.5	37.71	39.12	35.83	58.88	49.66	53.29	49.36	54.35	79.99	76.21	78.76	75.43	81.22
Stomach volume [mL]	27	24	29	37	33	80	76	79	81	78	241	216	220	219	201

**Table 3:** Horse stomach indexes.

Stomach Indexes [mm]															
Sex	M	M	M	F	F	M	M	M	F	F	M	M	F	F	F
Age [months]	4–5	4–5	4–5	4–5	4–5	7–8	7–8	7–8	7–8	7–8	10–11	10–11	10–11	10–11	10–11
Width-length	59.53	53.65	54.45	66.09	55.05	67.29	66.97	64.47	68.35	66.38	66.87	67.04	60.89	63.27	63.82
Thickness-length	58.48	51.93	62.80	65.58	57.11	56.60	57.53	59.50	56.36	60.71	53.00	55.90	56.68	53.42	57.15
Thickness-width	98.24	96.79	115.33	99.22	103.73	84.12	85.89	92.29	82.46	91.46	79.26	83.39	93.09	84.43	89.55
Curvatures	18.44	22.70	19.42	19.85	25.41	16.55	24.08	31.97	21.55	25.51	26.89	26.11	22.01	24.29	24.61
Cardia-pyloric	63.61	175.64	77.43	78.64	120.76	47.38	147.48	106.19	42.11	88.68	70.47	70.62	73.65	75.04	74.32

## Data Availability

Not applicable.
